# Delayed onset of striatal projection neuron hyperexcitability in *Fmr1−/y* mice

**DOI:** 10.3389/fncel.2025.1667476

**Published:** 2025-11-13

**Authors:** Lars Nelson, Michael Janeček, Michael Matarazzo, Yi-Chun Shih, Rui T. Peixoto

**Affiliations:** 1 Department of Psychiatry, University of Pittsburgh, Pittsburgh, PA, United States; 2 Center for Neuroscience at the University of Pittsburgh, Pittsburgh, PA, United States

**Keywords:** striatum, striatal projection neuron, neurodevelopment, Fragile X, *Fmr1*, autism spectrum disorder

## Abstract

**Introduction:**

Fragile X Syndrome (FXS), the most common genetic cause of intellectual disability and autism spectrum disorder (ASD), results from silencing of the *FMR1* gene and consequent loss of Fragile X Messenger Ribonucleoprotein (FMRP). FMRP deficiency disrupts neural development, leading to behavioral and motor deficits associated with striatal dysfunction. Although structural and functional abnormalities in striatal projection neurons (SPNs) have been observed in adult *Fmr1* knockout mice (*Fmr1−/y*), their developmental onset and contribution to early FXS pathophysiology remain unknown.

**Methods:**

We examined the postnatal maturation of SPNs in the dorsomedial striatum (DMS) of *Fmr1−/y* mice, assessing glutamatergic synaptic inputs and intrinsic excitability using whole-cell electrophysiology.

**Results:**

During postnatal development, *Fmr1* deficient SPNs display normal synaptic and intrinsic properties, consistent with typical maturation. In contrast, by P60, *Fmr1−/y* SPNs exhibit pronounced hyperexcitability in both dopamine D1 receptor–expressing SPNs (D1-SPNs) and D2 receptor–expressing SPNs (D2-SPNs), with more pronounced effects in D1-SPNs. Chronic aripiprazole treatment, a widely prescribed therapy for behavioral symptoms in FXS, fails to normalize SPN excitability, suggesting limited efficacy in addressing core SPN dysfunction.

**Discussion:**

These findings reveal that DMS SPN hyperexcitability in *Fmr1−/y* mice emerges after early postnatal development, pointing to a progressive trajectory of striatal abnormalities. In addition, these results underscore the importance of developmental timing in FXS pathophysiology and emphasize the need for targeted interventions to address SPN dysfunction.

## Introduction

Fragile X Syndrome (FXS), the most common inherited cause of intellectual disability and autism spectrum disorder (ASD), results from disruption of the 5′ untranslated region of the *FMR1* gene on the X chromosome (Richter and Zhao, 2021). This disruption typically involves a CGG trinucleotide repeat expansion, leading to hypermethylation and transcriptional silencing of *FMR1* and a consequent loss of Fragile X Messenger Ribonucleoprotein (FMRP). FMRP, an RNA-binding protein abundantly expressed in the brain, regulates the translation of numerous mRNA targets critical for synaptic plasticity and neural development (Darnell et al., 2011; Ascano et al., 2012). Its absence leads to a spectrum of behavioral and neurological impairments, including language delays, sensory hypersensitivity, irritability, hypotonia and a high prevalence of seizures, reflecting widespread neurological dysfunction (Bagni et al., 2012). Mouse models of FXS have provided critical insights into the role of FMRP in cortical circuit development and function, with loss of *Fmr1* inducing abnormal neuronal excitability, synaptic plasticity, and long-range connectivity (Hays et al., 2011; Haberl et al., 2015; Gibson et al., 2008; Pfeiffer and Huber, 2007; Martin et al., 2016). Many of these deficits emerge early in development, with patterns of abnormal cortical activity already observed during the first postnatal weeks (Gonçalves et al., 2013; Goel et al., 2018; Kourdougli et al., 2023). Recent studies have also observed deficits in striatal circuits in adult *Fmr1* knockout (KO) mice, suggesting a potential role for the striatum in the motor and behavioral symptoms of FXS (Longo et al., 2023; Mercaldo et al., 2023; Huebschman et al., 2022; Neuhofer et al., 2018; Neuhofer et al., 2015; Jung et al., 2012; Huebschman et al., 2020). However, whether striatal dysfunction emerges during early postnatal development remains unknown.

The striatum, the principal input structure of the basal ganglia, integrates diverse afferent inputs organized into distinct functional domains (Shepherd, 2013). The dorsal striatum is divided into the dorsomedial striatum (DMS), implicated in goal-directed behavior, and the dorsolateral striatum (DLS), associated with motor control. The ventral striatum, including the nucleus accumbens (NAc), mediates reward processing and emotional regulation. Deficits in these striatal functions align with core symptoms of autism (Fuccillo, 2016), and neuroimaging studies consistently reveal hypertrophy and altered connectivity of striatal regions in individuals with ASD (Wolff et al., 2013; Turner et al., 2006; Langen et al., 2014; Langen et al., 2009; Langen et al., 2007; Long et al., 2016; Ismail et al., 2016). Furthermore, striatal neurons exhibit one of the highest expression rates of ASD risk genes (Chang et al., 2015; Willsey et al., 2013). Many of these genes, particularly those associated with synaptic maturation and function, are dynamically regulated in the striatum during early postnatal development (Peixoto et al., 2019), further pointing to a convergence of ASD genetic risk in striatal circuit maturation. The activity of striatal circuits is primarily driven by glutamatergic input onto striatal projection neurons (SPNs), which express either dopamine D1 (D1-SPN) or D2 receptors (D2-SPN) (Shepherd, 2013). The functional balance between these two SPN populations is crucial for motor control and cognitive processes, and disruptions of their activity are linked to severe symptoms in a wide range of psychiatric and neurodevelopmental disorders, including ASD (Fuccillo, 2016; Benthall et al., 2018; Wang et al., 2017; Rothwell et al., 2014; Gittis and Kreitzer, 2012).

In adult *Fmr1−/y* mice, FMRP deficiency induces complex, region-specific alterations in striatal circuits. In the NAc, loss of FMRP impairs synaptic plasticity of glutamatergic synapses in SPNs (Neuhofer et al., 2018; Jung et al., 2012). Additionally, NAc SPNs of *Fmr1−/y* mice exhibit altered intrinsic properties, with opposing changes in membrane excitability and action potential dynamics in D1-SPNs versus D2-SPNs (Giua et al., 2023). These findings indicate a cell-type-specific role for FMRP in regulating properties of mature SPNs, with potential deleterious implications for striatal circuit function. In the DLS, the loss of FMRP alters dendritic structure and synaptic density (Longo et al., 2023). While an initial study reported subtle reductions in stubby dendritic spines across SPNs (Huebschman et al., 2022), subsequent analysis of separate SPN populations revealed a predominant increase in spine density in D1-SPNs, with no significant changes observed in D2-SPNs (Longo et al., 2023). In contrast, the effects of FMRP loss in the DMS remain poorly characterized, with the exception of one study showing no changes in dendritic spine density in DMS SPNs of adult *Fmr1−/y* mice (Huebschman et al., 2022). However, whether DMS SPNs exhibit other functional abnormalities remains unknown. This gap in knowledge is particularly significant, as structural deficits in the head of the caudate, analogous to the DMS in rodents, have been reported in individuals with FXS (Wolff et al., 2013) and are among the most recurrent findings in imaging studies of individuals with ASD (Turner et al., 2006; Langen et al., 2014; Langen et al., 2009; Langen et al., 2007; Long et al., 2016; Ismail et al., 2016). FMRP expression peaks in the striatum during perinatal periods (Gholizadeh et al., 2015), suggesting a critical role in the SPN maturation. Moreover, cortical dysfunction in *Fmr1−/y* mice emerges during the first postnatal week (Gonçalves et al., 2013; Goel et al., 2018; Kourdougli et al., 2023), a developmental period marked by strong reciprocal interactions between cortical and striatal circuits (Peixoto et al., 2019; Peixoto et al., 2016; Kozorovitskiy et al., 2015). Together, these observations raise the possibility that SPN dysfunction begins during early postnatal development.

Despite the high prevalence and significant disease burden of FXS, no prophylactic treatments have been developed to date (Jeste and Geschwind, 2016). Aripiprazole, an atypical antipsychotic, is commonly prescribed to manage irritability and aggression in individuals with FXS (Erickson et al., 2010). However, its efficacy in targeting pathophysiological mechanisms associated with FXS remains poorly understood. To address these gaps, we investigated the developmental trajectory of glutamatergic synaptic inputs and intrinsic excitability of DMS SPNs in *Fmr1−/y* mice across early postnatal (P14-P15) and adult (P60) stages. In addition, we assessed the potential for chronic aripiprazole treatment to ameliorate intrinsic SPN dysfunction in adult *Fmr1−/y* mice. We found that SPN hyperexcitability in the DMS emerges only after early postnatal development, affecting both D1- and D2-SPNs but with greater severity in D1-SPNs, and that chronic aripiprazole treatment failed to reverse these deficits. These results underscore the importance of developmental timing in FXS striatal pathophysiology and suggest that aripiprazole does not target core physiological abnormalities of SPNs.

## Methods

### Animals

All experimental manipulations on mice were performed in accordance with protocols approved by the Institutional Animal Use and Care Committee at the University of Pittsburgh in compliance with the guidelines described in the US National Institutes of Health *Guide for the Care and Use of Laboratory Animals*. Mice were housed on a 12/12 h light/dark cycle with chow and water provided ad libitum. Mice were weaned at P21-23 and separated by sex in cages of 2-5 animals of mixed genotypes. *Fmr1* mutant mice B6.129P2-*Fmr1^tm1Cgr^*/J and D1-Tom^+^ B6. Cg-Tg (Drd1a-tdTomato)^6Calak^/J were obtained from The Jackson Laboratory (#003025 and #016204). Genetic crosses were established between *Fmr1^+/−^* carrier dams and D1-Tom^+/−^ males to obtain D1-Tom^+^ *Fmr1+/y* and *Fmr1−/y* littermates. Characterization of neural properties by electrophysiology was performed in male *Fmr1+/y* and *Fmr1−/y* age-matched mice.

### Aripiprazole preparation and administration

Aripiprazole (Millipore-Sigma: 1042634) was dissolved at 10 mg/mL in 100% DMSO and stored in the dark at room temperature. The Aripiprazole/DMSO solution was dissolved in 1% Tween-80 in 0.9% (0.375 mg/mL final concentration) saline each day prior to injections. Vehicle solution contained DMSO (0.0375 mL/mL) and 1% Tween-80 in 0.9% saline. Mice were injected with 3 mg/kg of Aripiprazole or vehicle intraperitoneally. Mice were weighed on day 1 before the first injection and the injection volume was adjusted for weight. Adult mice were administered Aripiprazole for 14 days every afternoon. The day after the final injection (~18 h later), tissue was collected for acute slice electrophysiology.

### Brain slice preparation and whole-cell electrophysiology

Acute brain slices were prepared following anesthesia by isoflurane inhalation and transcardiac perfusion with ice-cold artificial cerebrospinal fluid (ACSF) containing (in mM): 125 NaCl, 2.5 KCl, 25 NaHCO_3_, 2 CaCl_2_, 1 MgCl_2_, 1.25 NaH_2_PO_4_ and 25 glucose (310 mOsm per kg). Cerebral hemispheres were removed and transferred into a slicing chamber containing ice-cold ACSF. Coronal slices including ACC (275 μm thick) were cut with a Leica VT1200s vibratome and transferred for 10 min to a holding chamber containing choline-based solution consisting of (in mM): 110 choline chloride, 25 NaHCO_3_, 2.5 KCl, 7 MgCl_2_, 0.5 CaCl_2_, 1.25 NaH_2_PO_4_, 25 glucose, 11.6 ascorbic acid, and 3.1 pyruvic acid at 33 °C. Slices were subsequently transferred to a chamber with pre-warmed ACSF (33 °C) and gradually cooled down to room temperature (20–22 °C). All recordings were obtained within 4 h of slicing. Both ACSF and choline solution were constantly bubbled with 95% O_2_ and 5% CO_2_. Individual slices were transferred to a recording chamber mounted on an upright microscope (Scientifica SliceScope with Olympus optics) and continuously perfused (1–2 mL per minute). Cells were visualized using a 40 × water-immersion objective with infrared illumination. Whole-cell voltage clamp recordings were made from SPNs in the dorsomedial striatum. Recording electrode pipettes (3-4 MΩ) pulled from borosilicate glass (BF150-86-7.5, Sutter Instruments). Voltage-clamp recordings were performed in ACSF at room temperature (20–22 °C) with a Cs^+^-based internal solution containing (in mM): 130 CsMeSO_4_, 10 HEPES, 1.8 MgCl_2_, 4 Na_2_ATP, 0.3 NaGTP, and 8 Na_2_-phosphocreatine,10 CsCl_2,_ 3.3 QX-314 (Cl^−^ salt), (pH 7.3 adjusted with CsOH; 300 mOsm per kg). In voltage-clamp experiments, errors due to voltage drop across the series resistance (<20 MΩ) were left uncompensated. For mEPSC recordings, ACSF contained 1 μM TTX, 1 μM (RS)-CPP, and 1 μM Gabazine, and recordings were performed with V_m_ = −70 mV. After breaking in, cells were left to stabilize for 4 min and currents were then acquired continuously for 5 min. Membrane currents and potentials were amplified and low-pass filtered at 3 kHz using Multiclamp 700B amplifier (Molecular Devices), digitized at 10 kHz, and acquired using National Instruments acquisition boards and a custom version of ScanImage written in MATLAB (Mathworks). Calculation of input resistance and membrane capacitance in voltage clamp recordings was performed by fitting evoked currents in response to −5 mV voltage steps in the first seconds after cell break-in. Current-clamp recordings were performed in ACSF near physiological temperature (31–33 °C) using a potassium-based internal solution containing (in mM): 130 KMeSO_3_, 10 HEPES, 3 KCl, 1 EGTA, 4 Na_2_ATP, 0.3 NaGTP, and 8 Na_2_-phosphocreatine, (pH 7.3 adjusted with KOH; 300 mOsm per kg). The junction potential (−9 mV) was left uncompensated. Cell was broken in with V_m_ = −70 mV and allowed to stabilize for 4 min in voltage-clamp. After stabilization, resting membrane potential was measured at I = 0. Current-clamp recording was performed by adjusting holding current to maintain the V_m_ at −75 mV. For each experiment four cycles of 300 ms baseline, 700 ms current injection step and a 2000 ms baseline ending the acquisition. For the adult timepoints the following current steps were used: −100, −50, −25, 0, 25, 50, 100, 150, 200, 250, 300, 350, 400. For the P14-P15 timepoint the following current steps were used: −100, −75, −50, −25, 0, 25, 50, 75, 100, 125, 150, 175, 200. For the aripiprazole experiment the following current steps were used: −100, −75, −50, −25, 0, 25, 50, 75, 100, 125, 150, 175, 200. Recording data was saved as Matlab files for subsequent off-line analysis.

### Electrophysiology data analysis

Adult D1/D2 mEPSCs were analyzed using a custom program in Igor Pro. For all other experiments mEPSCs and current-clamp traces were analyzed using a custom python-based program ClampSuite available at https://github.com/LarsHenrikNelson/ClampSuite. For mEPSC analysis, acquisition offset was removed by subtracting the mean. Recordings were filtered using a zero-phase Remez filter with a low pass filter at 600/300 Hz. Events were identified by FFT deconvolution. Tau was estimated as the time when the trace reached 37% of peak amplitude. Rise time was calculated as the time from the baseline start of the mEPSC to the peak. Rise rate is the amplitude of the peak divided by the rise time. mEPSC events were excluded based on the following criteria: Amplitude lower than 7 pA. For all experiments cells were excluded if the access resistance raised above 20 MOhm during the recording. For adult mEPSC we excluded cells if they had a mEPSC frequency above 7 hertz, a membrane resistance below 120 mOhm and a capacitance greater than 60 pF or a mEPSC decay tau <3.5. For P14-P15 mEPSC recordings we excluded based on mEPSC decay tau < 3.5. For current-clamp analysis we excluded neurons with a drop in peak AP voltage over the duration of the current step greater than 15 mV, and with a peak AP voltage lower than 30 mV. Interneurons were excluded based on half-width and firing frequency. Interneuron half-width was ~½ of a SPN half-width and the firing would exceed 100 Hertz. Rheobase was defined as the lowest current step that triggered at least one action potential. The AP threshold was identified using the 3^rd^ derivative. The I-V curve was calculated by fitting a regression between current step amplitude and delta-V for the first 6 current steps. A sigmoid curve was fit to the adult D1/D2 FI curves to determine whether FI curve differences were due to change in slope, maximum firing rate or pA offset (similar to rheobase) using the follow equation: $\frac{1}{1+e^{\frac{x-A}{-B}}}\cdot C+D$ , where *A* is the pA offset, *B* is the slope, *C* is the maximum firing rate and *D* is the firing rate offset. The timing of AP waveform features was the time from spike threshold to the AP feature.

### Statistical analyses

Two-way ANOVA was performed for comparing parameters affected by two factors. *p* < 0.05 was used as the significance threshold. Multiple comparisons were run using a Welch’s test followed by a Holm *p*-value correction. All plots show mean +/− SEM. Each shape in a plot group (i.e., *Fmr1−/y* x D1-SPN) represents a unique mouse for that group. η^2^ and the 95% confidence intervals of the difference were reported. ω^2^ is reported in Supplementary Table 1. Confidence intervals using an alpha = 0.05 for multiple comparisons were also reported. For all of the statistics see the Supplementary Table 1. Statistical calculations were performed using *StatsModels* (Python) or for FI curves *afex*, *effectsize* and *emmeans* (R). For the FI curve current injection step was considered a within subject factor and genotype and subtype as a between subject factor. For the FI curves η^2^ was the partial-η^2^ and ω^2^ was partial-ω^2^. Specific statistical analyses are detailed in the respective figure legends or results section for each dataset. For the P14-P15 mEPSC experiment a total of 10 D1-SPN and 11 D2-SPN cells from 6 *Fmr1+/y* mice and 12 D1-SPN and 12 D2-SPN cells from 5 *Fmr1−/y* mice were analyzed. For the P14-P15 current clamp experiment, a total of 20 D1-SPN and 18 D2-SPN cells from 4 *Fmr1+/y* mice and 11 D1-SPN and 14 D2-SPN cells from 4 *Fmr1−/y* mice were analyzed. For the adult D1/D2 mEPSC experiment, a total of 23 D1-SPN and 28 D2-SPN cells from 4 mice and 30 D1-SPN and 24 D2-SPN cells from 5 mice were analyzed. For the adult D1/D2 current clamp experiment, a total of 24 D1-SPN and 16 D2-SPN cells from 5 *Fmr1+/y* mice and 27 D1-SPN and 14 D2-SPN from 6 *Fmr1−/y* mice were analyzed. For the adult aripiprazole current clamp experiment, a total of 22 cells from 3 *Fmr1+/y* x Aripiprazole mice, 28 cells from 3 *Fmr1+/y* x Vehicle mice, 29 cells from 3 *Fmr1−/y* x Aripiprazole mice, 26 cells from 3 *Fmr1−/y* x Vehicle mice were analyzed.

## Results

### Normal glutamatergic synaptic input in DMS D1- and D2-SPNs of *Fmr1−/y* mice at P14-P15

**Figure 1:**
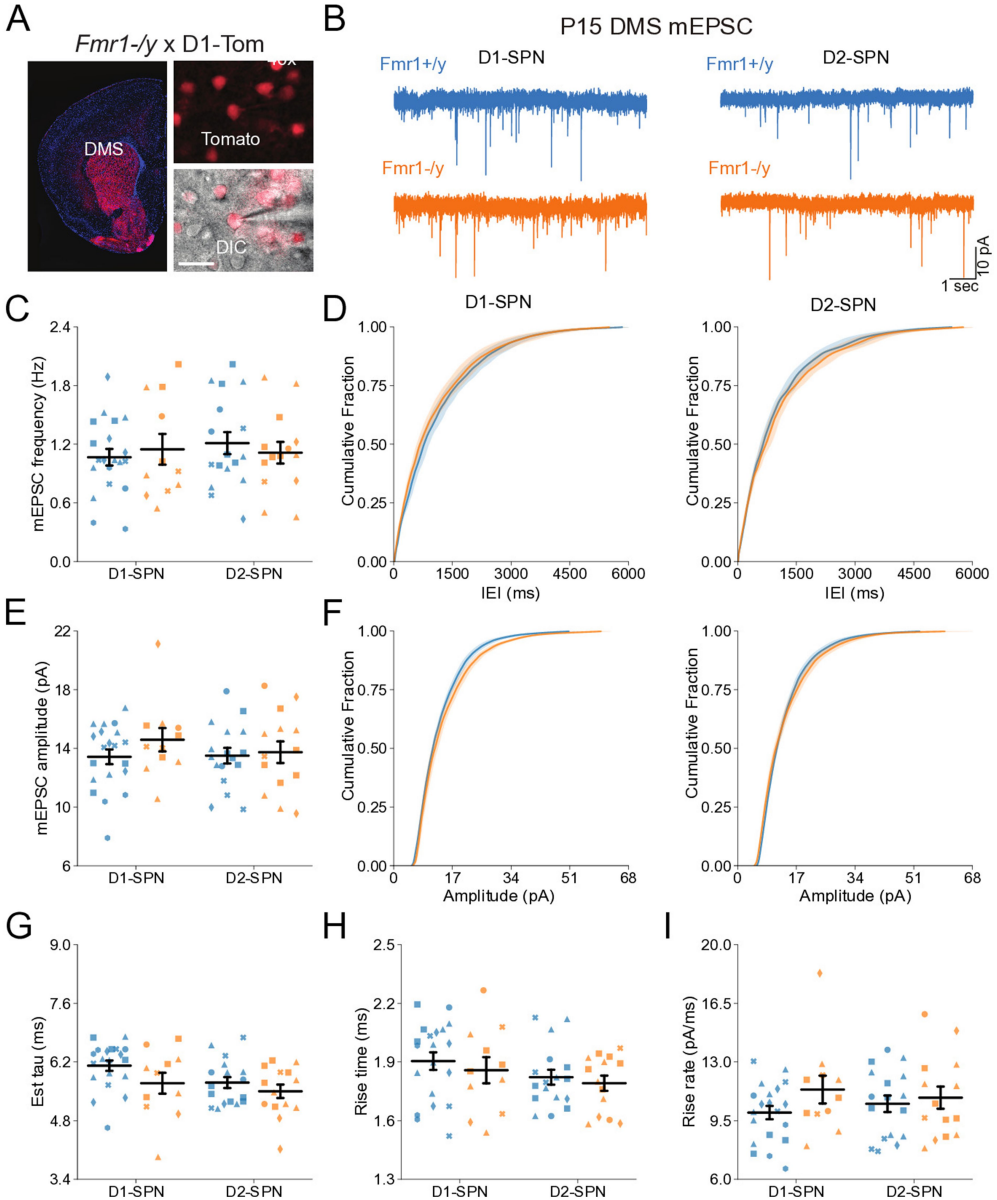
Normal synaptic transmission in D1- and D2-SPNs of the DMS in P14-P15 *Fmr1−/y* mice. **(A)** Schematic representing a coronal brain section and whole-cell recordings of D1-SPNs (labeled with D1-Tomato) and D2-SPNs (td-Tomato negative) in the DMS of P14-P15 *Fmr1−/y* and *Fmr1+/y* mice. **(B)** Representative AMPAR-mediated mEPSCs in D1-SPNs (left) and D2-SPNs (right) of *Fmr1+/y* (blue) and *Fmr1−/y* (orange) mice. n = 10, 11 D1- and D2-SPN from N = 6 *Fmr1+/y* mice; n = 12, 12 D1- and D2-SPN from N = 5 *Fmr1−/y* mice **(C)** Average mEPSC frequency. **(D)** Cumulative distribution of inter-event intervals (IEIs) of mEPSCs in D1- and D2-SPNs (left and right, respectively). **(E)** Average mEPSC amplitude. **(F)** Cumulative distribution of mEPSC amplitudes in D1- and D2-SPNs (left and right respectively). **(G)** Average mEPSC decay tau, **(H)** rise time and **(I)** rise rate. Plots show individual data points, with shape representing a mouse within the group (i.e., *Fmr1−/y* x D1-SPN) and the summary with mean ± SEM. *p < 0.05, **p < 0.01, ***p < 0.005, ****p < 0.001.

To determine whether *Fmr1* deletion affects the early postnatal development of glutamatergic synapses onto DMS D1- and D2-SPNs, we recorded AMPAR-mediated miniature excitatory postsynaptic currents (mEPSCs) in acute brain slices from male *Fmr1+/y* or *Fmr1−/y* mouse pups carrying a *Drd1a-tdTomato* allele. Recordings were performed at P14-P15, a developmental stage marked by rapid maturation of SPNs and analogous to early infancy in humans, when ASD symptoms typically begin to emerge (Peixoto et al., 2016; Lord et al., 2000). Expression of tdTomato was used for identification of D1-SPNs (Figure 1A). Notably, the tdTomato-negative cell population includes striatal interneurons, but these represent a small fraction of total striatal neurons (Tepper and Bolam, 2004) and we further excluded putative interneurons from analysis based on mEPSC kinetics (See methods). In addition, a small proportion (~5%) of SPNs in dorsal striatum have been reported to co-express both D1 and D2 receptors (Biezonski et al., 2015). Although this dual expression may introduce a minor margin of error in classification, the vast majority of SPNs segregate into D1 or D2 receptor-expressing populations. For the purposes of this study, we therefore refer to tdTomato-positive neurons as D1-SPNs and tdTomato-negative neurons as D2-SPNs. To measure AMPAR mEPSCs, we performed whole-cell recordings in the presence of the voltage-gated sodium channel blocker tetrodotoxin (TTX) with membrane potential clamped at -70 mV. Quantification of mEPSC frequency (Figures 1C,D) or amplitude (Figures 1E,F) revealed no significant difference between genotypes in either D1- or D2-SPNs (See Supplementary Table 1 for detailed values and statistical results). To further assess potential differences in synaptic AMPAR function, we analyzed mEPSC decay kinetics. Overall, D2-SPNs exhibited faster decay kinetics relative to D1-SPNs (Figure 1G; see Supplementary Table 1 for all SPN subtype comparison statistics). Although there was a trend toward faster mEPSC decay in *Fmr1−/y* relative to *Fmr1+/*y SPNs, this difference was not statistically significant (Figure 1G), and no genotype differences were observed in mEPSC rise time (Figure 1H) or rise rate (Figure 1I). Together, these findings suggest that *Fmr1* deletion does not substantially alter glutamatergic synapse number or postsynaptic AMPAR function in DMS D1-SPNs or D2-SPNs at P14-P15.

### Normal intrinsic excitability and passive membrane properties of DMS D1- and D2-SPNs of *Fmr1−/y* mice at P14-P15

**Figure 2:**
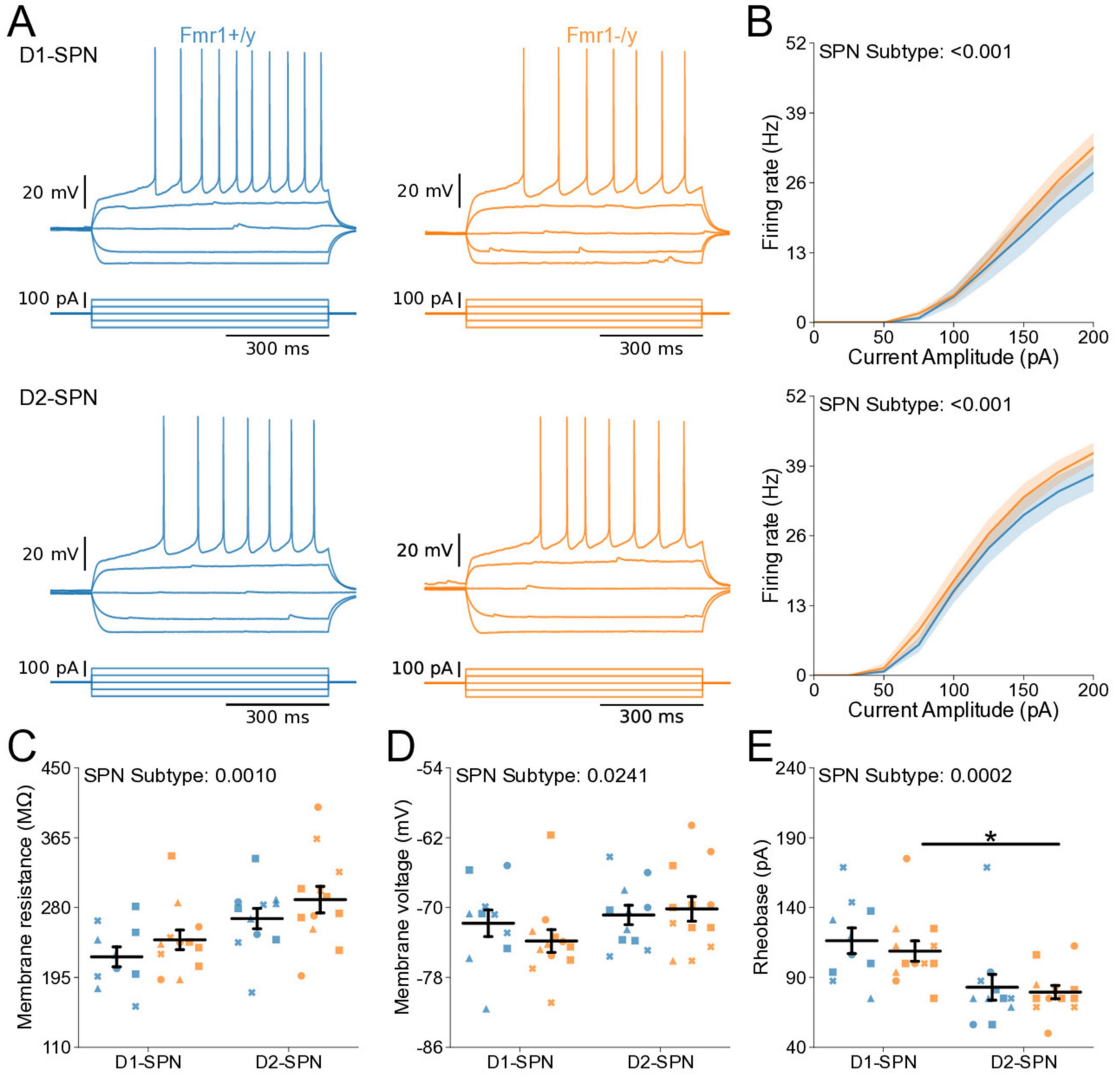
Normal intrinsic excitability and passive membrane properties of D1- and D2-SPNs in P14-P15 *Fmr1−/y* mice. **(A)** Representative current-clamp recordings showing action potential firing in response to current injections in D1-SPNs (top) and D2-SPNs (bottom) of *Fmr1−/y* (orange) and *Fmr1+/y* (blue) P-15 mice. n = 20, 18 D1- and D2-SPN cells from N = 4 *Fmr1+/y* mice; n = 11, 14 D1- and D2-SPN cells from N = 4 *Fmr1−/y* mice **(B)** Input–output relationship of firing frequency versus current amplitude (I-F curves) in D1- and D2-SPNs (top and bottom respectively). **(C)** Average membrane resistance in D1- and D2-SPNs. **(D)** Average resting membrane potential in D1- and D2-SPNs. **(E)** Average rheobase current in D1- and D2-SPNs. Plots show individual data points, with shape representing a mouse within the group (i.e., *Fmr1−/y* x D1-SPN) and the summary with mean ± SEM. *p < 0.05, **p < 0.01, ***p < 0.005, ****p < 0.001 for multiple comparisons.

DMS SPNs undergo extensive maturation of their intrinsic properties during postnatal development (Peixoto et al., 2016; Tepper et al., 1998). To determine whether *Fmr1* deletion impacts the excitability and passive membrane properties of D1- and D2-SPNs during this period, we performed current-clamp whole-cell recordings at P14-P15. Recordings were performed at near-physiological temperature using a potassium-based internal solution to measure membrane voltage changes in response to stepped current injections (Figure 2A). ANOVA revealed no main effects of genotype but identified effects of SPN subtype on several excitability measures (I–F, membrane resistance, RMP, rheobase; Figures 2B–E), pointing to increased excitability of D2-SPNs. Thus, at P14–P15 we found no evidence that *Fmr1* deletion alters intrinsic or passive membrane properties in either SPN subtype.

### Normal action potential properties of DMS D1- and D2-SPNs of *Fmr1−/y* mice at P14-P15

**Figure 3:**
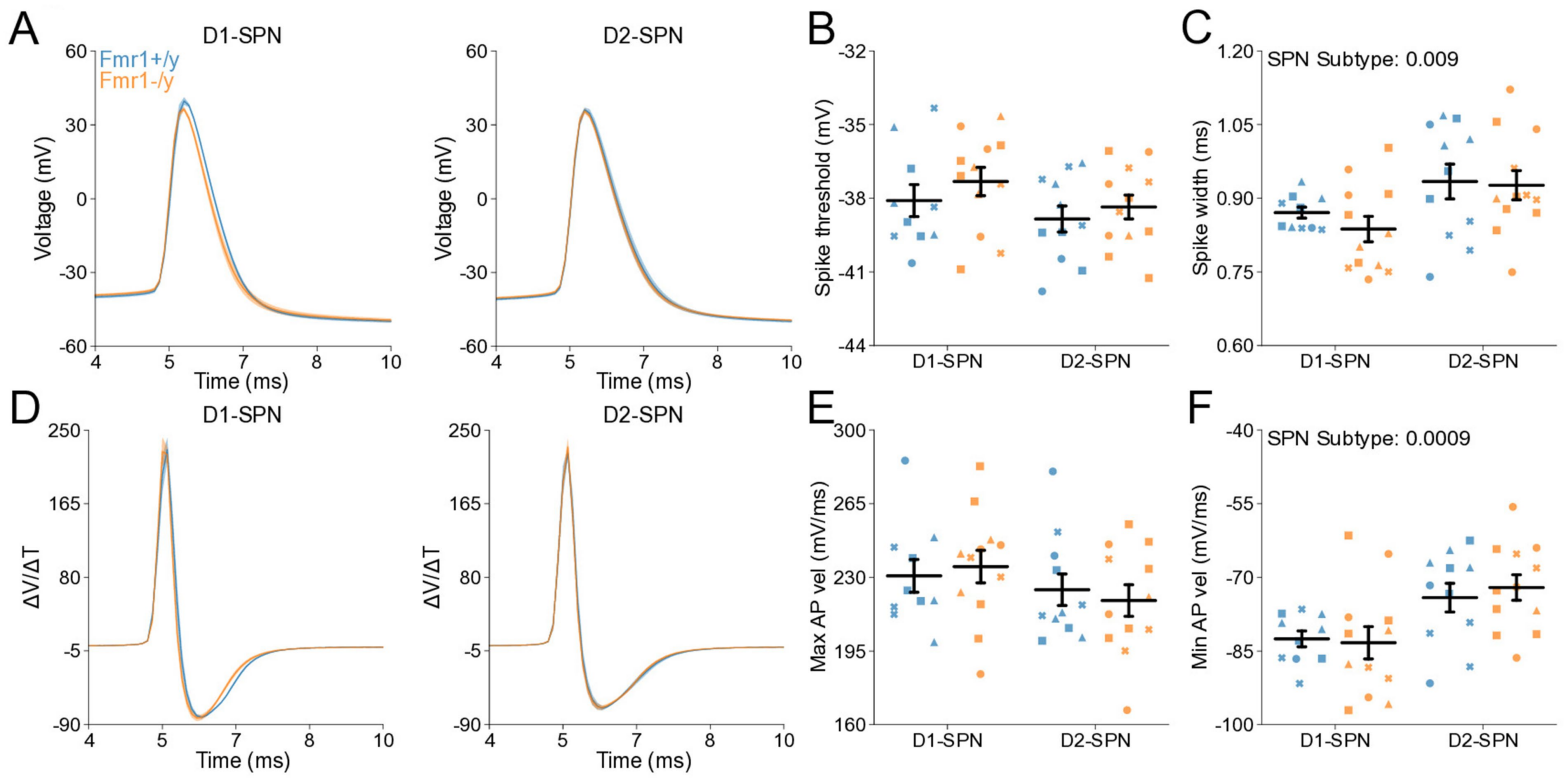
Action potential properties in D1- and D2-SPNs of P14-P15 *Fmr1−/y* mice. **(A)** Representative single action potential traces recorded from D1-SPNs (left) and D2-SPNs (right) of *Fmr1−/y* (orange) and *Fmr1+/y* (blue) P-15 mice. n = 20, 18 D1- and D2-SPN cells from N = 4 *Fmr1+/y* mice; n = 11, 14 D1- and D2-SPN cells from N = 4 *Fmr1−/y* mice **(B)** Average AP threshold in D1- and D2-SPNs. **(C)** Average AP half-width in D1- and D2-SPNs. **(D)** AP velocity (∆V/∆t) in D1- and D2-SPNs (left and right respectively). **(E)** Maximum AP velocity. **(F)** Minimum AP velocity. Plots show individual data points, with shape representing a mouse within the group (i.e., *Fmr1−/y* x D1-SPN) and the summary with mean ± SEM. *p < 0.05, **p < 0.01, ***p < 0.005, ****p < 0.001.

Deletion of *Fmr1* alters action potential (AP) kinetics of adult NAc SPNs (Giua et al., 2023). To investigate whether *Fmr1* deletion similarly affects APs of DMS D1- and D2-SPNs during postnatal development, we analyzed AP waveforms from rheobase traces from our current-clamp recordings (Figures 3A,D). AP threshold was similar across SPN subtypes and unaffected by genotype (Figure 3B). ANOVA revealed a main effect of SPN subtype on AP half-width, although *post hoc* comparisons did not reach significance within either genotype (Figure 3C). No main effects of SPN subtype or genotype were detected in the peak AP velocity (Figures 3D,E), the time of peak AP velocity (Supplementary Figure S1A), or the time of minimum AP velocity (Supplementary Figure S1B). Minimum AP velocity showed a main effect of SPN subtype, with slower values in D2-SPNs, although this difference did not reach significance in *post hoc* comparisons (Figure 3F). There was a main genotype effect on AP peak voltage, which was lower in *Fmr1−/y* SPNs compared to *Fmr1+/y* (Supplementary Figure S1C; Genotype: *p* = 0.0144, η^2^ = 0.121, 95% CI [0.531, 4.53]; SPN subtype: *p* = 0.0243, η^2^ = 0.101, 95% CI [0.316, 4.31]), with post hoc comparisons suggesting a stronger trend in D1-SPNs than in D2-SPNs (Supplementary Figure S1C; *Post hoc* comparison; D1-SPNs: *p* = 0.0902, 95% CI [0.571, 6.63]; D2-SPN: *p* = 0.616, 95% CI [−1.444, 4.351]). Finally, we found no genotype or SPN subtype differences in the afterhyperpolarization (AHP) amplitude (Supplementary Figure S1D), but observed a SPN subtype difference in the time of peak AHP in *Fmr1−/y* mice, driven by faster AHP kinetics in D1-SPNs (Supplementary Figure S1E; SPN subtype: *p* = 0.00377, η^2^ = 0.18, 95% CI [−0.246, −0.0508]; *Post hoc* comparison; *Fmr1+/y*: *p* = 0.295, 95% CI [−0.244, 0.041]; *Fmr1−/y*: *p* = 0.034, 95% CI [−0.335, −0.054]). These results indicate that *Fmr1* deletion does not significantly affect the AP waveform of DMS SPNs at P14-P15 with the exception of a modest reduction in AP peak voltage.

### Normal glutamatergic synaptic input in DMS D1- and D2-SPNs of adult *Fmr1−/y* mice

**Figure 4:**
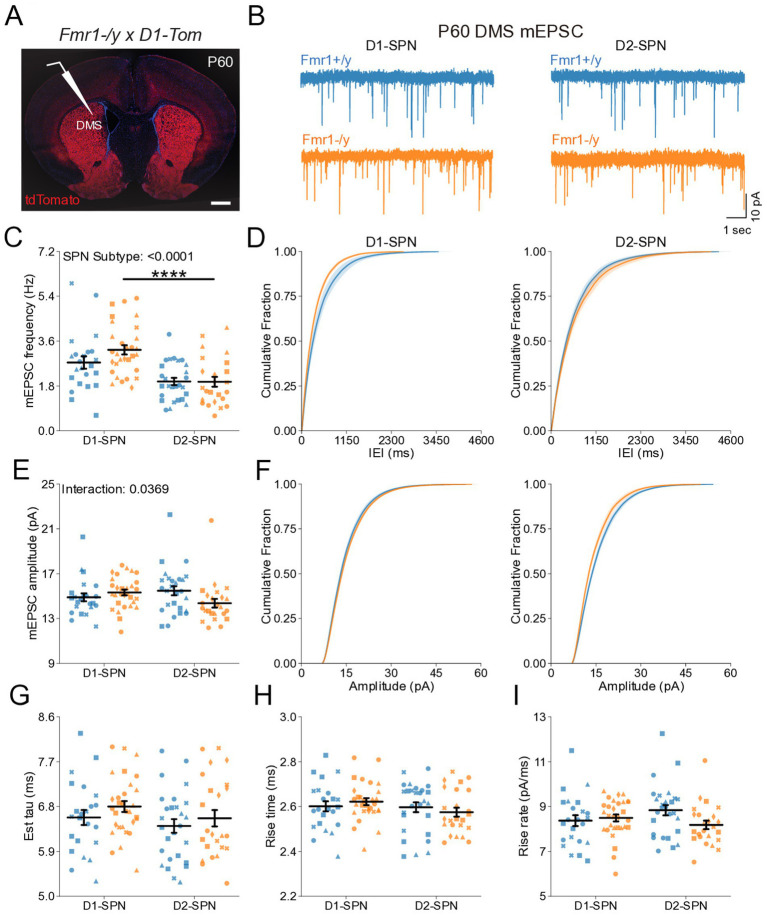
Normal glutamatergic synaptic transmission in D1- and D2-SPNs of the DMS in adult (P60) *Fmr1−/y* mice. **(A)** Schematic representing a coronal brain section and whole-cell recordings of D1-SPNs (labeled with D1-Tomato) and D2-SPNs in the DMS of P60 *Fmr1−/y* and *Fmr1+/y* mice. **(B)** Representative AMPAR-mediated mEPSCs in D1-SPNs (left) and D2-SPNs (right) of *Fmr1−/y* (orange) and *Fmr1+/y* (blue) mice. n = 23, 28 D1- and D2-SPNs from N = 4 *Fmr1+/y* mice; n = 30, 24 D1- and D2-SPNs from N = 5 *Fmr1−/y* mice **(C)** Average mEPSC frequency. **(D)** Cumulative distribution of inter-event intervals (IEIs) of mEPSCs in D1- and D2-SPNs (left and right respectively). **(E)** Average mEPSC amplitude. **(F)** Cumulative distribution of mEPSC amplitudes in D1- and D2-SPNs (left and right, respectively). **(G)** Average mEPSC decay tau, **(H)** rise time and **(I)** rise rate. Plots show individual data points with shape representing a mouse within the group (i.e., *Fmr1−/y* x D1-SPN) and the summary with mean ± SEM. *p < 0.05, **p < 0.01, ***p < 0.005, ****p < 0.001.

Previous studies have shown region-specific changes in dendritic spine density and morphology in SPNs of the NAc and DLS in adult *Fmr1−/y* mice (Longo et al., 2023; Huebschman et al., 2022). Given our findings during postnatal development, where no mEPSC deficits were detected (Figure 1), we sought to determine whether *Fmr1* loss leads to abnormal mEPSC frequency or amplitude in DMS D1- and D2-SPNs at P60 (Figure 4A). mEPSC frequency did not differ between genotypes overall, but was elevated in D1-SPNs compared to D2-SPNs (Figures 4C,D; SPN subtype: *p* < 0.0001, η^2^ = 0.168, 95% CI [0.541, 1.36], post hoc comparison; *Fmr1+/y*: *p* = 0.168; *Fmr1−/y*: p < 0.0001). In addition, we observed no genotype or SPN subtype differences in mEPSC peak amplitude (Figures 4E,F), decay tau (Figure 4G), rise time (Figure 4H) or rise rate (Figure 4I). Taken together, these findings suggest that *Fmr1* deletion has limited effects on AMPAR-mediated mEPSCs in DMS SPNs of adult mice.

### Hyperexcitability of DMS D1- and D2-SPNs in P60 *Fmr1−/y* mice

**Figure 5:**
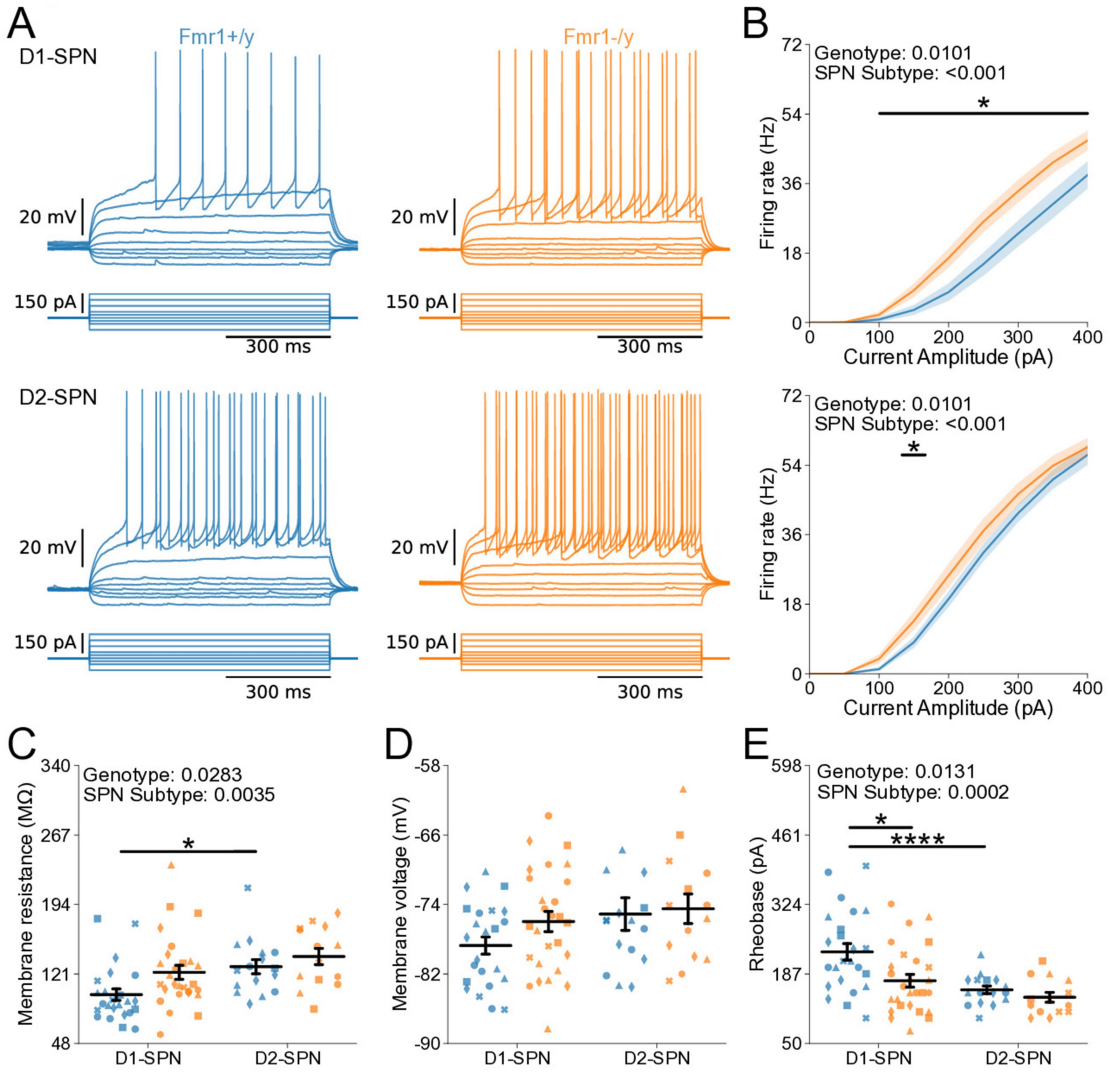
Increased intrinsic excitability of D1- and D2-SPNs in adult P60 *Fmr1−/y* mice. **(A)** Representative current-clamp recordings showing action potential firing in response to current injections in D1-SPNs (top) and D2-SPNs (bottom) of *Fmr1+/y* (blue) and *Fmr1−/y* (orange) P60 mice. n = 24, 16 D1- and D2-SPNs from N = 5 *Fmr1+/y* mice; n = 27, 14 D1- and D2-SPNs from N = 6 *Fmr1−/y* mice. **(B)** Input–output relationship of firing frequency versus current amplitude (I-F curves) in D1- and D2-SPNs (top and bottom respectively). **(C)** Average membrane resistance. **(D)** Average resting membrane potential. **(E)** Average rheobase current. Plots show individual data points, with shape representing a mouse within the group (i.e., *Fmr1−/y* x D1-SPN) and the summary with mean ± SEM. *p < 0.05, **p < 0.01, ***p < 0.005, ****p < 0.001.

We further characterized the intrinsic properties of DMS SPNs in adult *Fmr1−/y* mice by performing whole-cell current-clamp recordings as previously described (Figure 2). Notably, we detected a pronounced increase in the I-F relationship in both D1- and D2-SPNs of *Fmr1−/y* mice, with a larger effect observed in D1-SPNs (Figure 5B; Genotype: *p* = 0.0101, η^2^ = 0.0828; SPN subtype: *p* < 0.001, η^2^ = 0.215; Pulse Amplitude: *p* < 0.001, η^2^ = 0.891; Genotype*Pulse Amplitude: *p* < 0.001, η^2^ = 0.0615; SPN subtype*Pulse Amplitude: *p* < 0.001, η^2^ = 0.211). Multiple comparisons showed that in D1-SPNs the firing rate was increased in *Fmr1−/y* compared to *Fmr1+/y* at 100-400 pA current injection steps while the firing rate in D2-SPNs was only increased at the 150 pA step (See supplementary Table 1 for detailed RM ANOVA results). We fit each SPN F–I curve with a sigmoid function and found that the estimated maximum firing rate was elevated in D2-SPNs relative to D1-SPNs in both genotypes (Supplementary Figure S2B), whereas the slope was significantly lower in D1-SPNs predominantly in *Fmr1+/y* animals (Supplementary Figure S2A). However, no genotype differences were detected. The current amplitude offset was reduced in *Fmr1−/y* SPNs compared to *Fmr1+/y*, with a stronger effect in D1-SPNs (Supplementary Figure S2C; Genotype: *p* = 0.0008, η^2^ = 0.117, 95% CI [15.5, 56.8]; *Post hoc* comparison D1-SPNs: *p* = 0.0038, 95% CI [19.97, 77.05]; D2-SPN: *p* = 0.124, 95% CI [−1.318, 48.81]; SPN subtype: *p* = 0.0004, η2 = 0.13, 95% CI [17.6, 58.8]; Post hoc comparisons: *Fmr1+/y: p* = 0.0012, 95% CI [24.82, 76.35]; *Fmr1−/y*: p = 0.124, 95% CI [−2.01, 53.65]. Membrane resistance showed main effects of genotype and SPN subtype and was elevated in *Fmr1−/y* mice and D2-SPNs, respectively (Figure 5C; Genotype: *p* = 0.0283, η2 = 0.0544, 95% CI [−32.2, −1.86]; SPN subtype: *p* = 0.00351, η2 = 0.0989, 95% CI [−38.2, −7.79]). There was no genotype or SPN subtype effect on resting membrane potential (Figure 5D). Consistent with neural hyperexcitability and shifted I-F relationship, *Fmr1−/y* D1-SPNs exhibited lower rheobase currents compared to *Fmr1+/y*, with no statistically significant difference observed in D2-SPNs after multiple comparisons (Figure 5E; Genotype: *p* = 0.0131, η^2^ = 0.0644, 95% CI [7.73, 63.9]; SPN subtype: *p* = 0.000284, η^2^ = 0.144, 95% CI [25.6, 81.7]; Post hoc comparison; D1-SPNs: *p* = 0.0248, 95% CI [15.4, 98.4]); D2-SPNs: *p* = 0.0937, 95% CI [0.475, 64.6]). These results indicate that *Fmr1* deletion increases DMS SPN excitability in adult mice, with more pronounced effects in D1-SPNs.

### Altered action potential properties of DMS D1- and D2-SPNs in P60 *Fmr1−/y* mice

**Figure 6:**
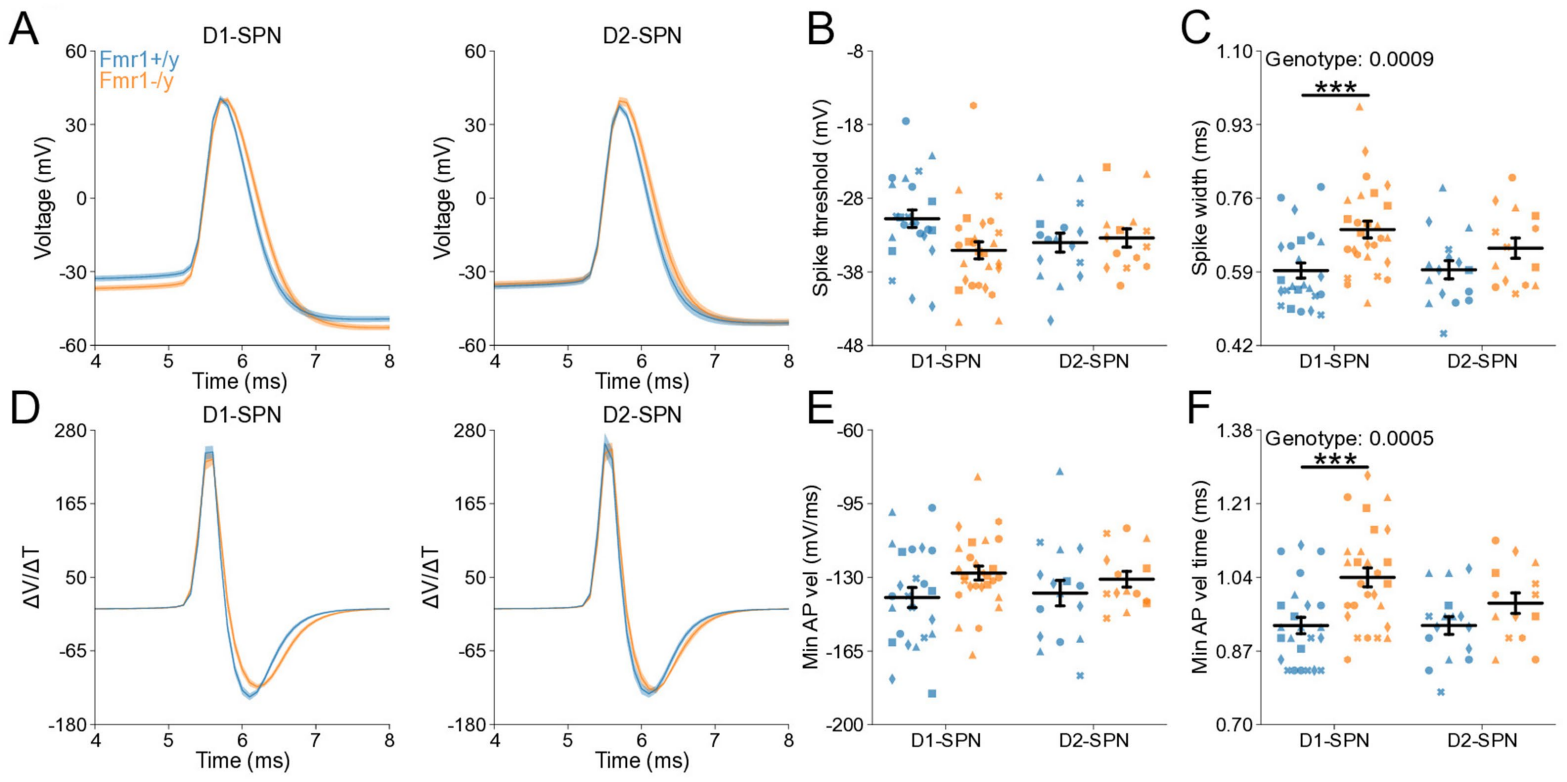
Action potential properties in D1- and D2-SPNs of adult P60 *Fmr1−/y* mice. **(A)** Representative single action potential traces recorded from D1-SPNs (left) and D2-SPNs (right) of *Fmr1−/y* (orange) and *Fmr1+/y* (blue) P60 mice. n = 24, 16 D1- and D2-SPNs from N = 5 *Fmr1+/y* mice; n = 27, 14 D1- and D2-SPNs from N = 6 *Fmr1−/y* mice. **(B)** AP threshold potential and **(C)** AP half-width in D1- and D2-SPNs. **(D)** AP velocity (∆V/∆t) (left and right respectively). **(E)** Minimum AP velocity. **(F)** Time of the minimum AP velocity. Plots show individual data points, with shape representing a mouse within the group (i.e., *Fmr1−/y* x D1-SPN) and the summary with mean ± SEM. *p < 0.05, **p < 0.01, ***p < 0.005, ****p < 0.001.

To determine whether changes in membrane resistance and rheobase observed in P60 *Fmr1−/y* SPNs are associated with abnormal AP properties, we analyzed AP kinetics from whole-cell current-clamp recordings (Figures 6A,D). Analysis of AP threshold did not reveal a statistically significant interaction between genotype and SPN subtype (Figure 6B). There was a main genotype effect on AP half-width, with broader APs in *Fmr1−/y* SPNs compared to *Fmr1+/y*, and post hoc analyses suggested a stronger effect in D1-SPNs than in D2-SPNs (Figure 6C; Genotype: *p* = 0.000936, η^2^ = 0.13, 95% CI [−0.114, −0.0304]; Post hoc comparison; D1-SPNs: *p* = 0.00276, 95% CI [0.0421, 0.147]; D2-SPNs: *p* = 0.372, 95% CI [−0.014, 0.114]). We found no genotype difference in peak AP velocity (Supplementary Figure S2D) but observed a delayed time of peak AP velocity in *Fmr1−/y* compared to *Fmr1+/y*, predominantly in D1-SPNs (Supplementary Figure S2E; Genotype: *p* = 0.0395, η^2^ = 0.0513, 95% CI [−0.0318, −0.0008]); Post hoc comparison D1-SPNs: *p* = 0.0319, 95% CI [0.00764, 0.0476]; D2-SPNs: *p* = 1.0, 95% CI [−0.016, 0.0260]. There was no significant genotype effect in minimum AP velocity (Figure 6E; Genotype: *p* = 0.0545, η^2^ = 0.047, 95% CI [−1.84, 0.0181]). However, the time of minimum AP velocity was delayed in *Fmr1−/y* SPNs relative to *Fmr1+/y,* with post hoc analyses indicating stronger effects in D1-SPNs (Figure 6F; Genotype: *p* = 0.00055, η^2^ = 0.139, 95% CI [−0.126, −0.0364]; Post hoc comparison D1-SPNs: *p* = 0.00143, 95% CI [0.0529, 0.169]; D2-SPNs: *p* = 0.222, 95% CI [−0.012, 0.115]). AP peak voltage (Supplementary Figure S2F) and peak AHP amplitude were not altered by loss of *Fmr1* (Supplementary Figure S2D) but the time of peak AHP was delayed in the *Fmr1−/y* group, with longer delay in D1-SPNs (Supplementary Figure S2H; Genotype: *p* = 0.00234, η^2^ = 0.0109, 95% CI [0.0518 0.23]; Post hoc comparison D1-SPNs: *p* = 0.0102, 95% CI [0.0647, 0.287]; D2-SPNs: *p* = 0.260, 95% CI [−0.033, 0.245]). These findings indicate that *Fmr1* deletion alters AP properties of mature DMS SPNs, with stronger effects in D1-SPNs.

### Chronic aripiprazole treatment does not normalize DMS SPN hyperexcitability in *Fmr1−/y* mice

**Figure 7:**
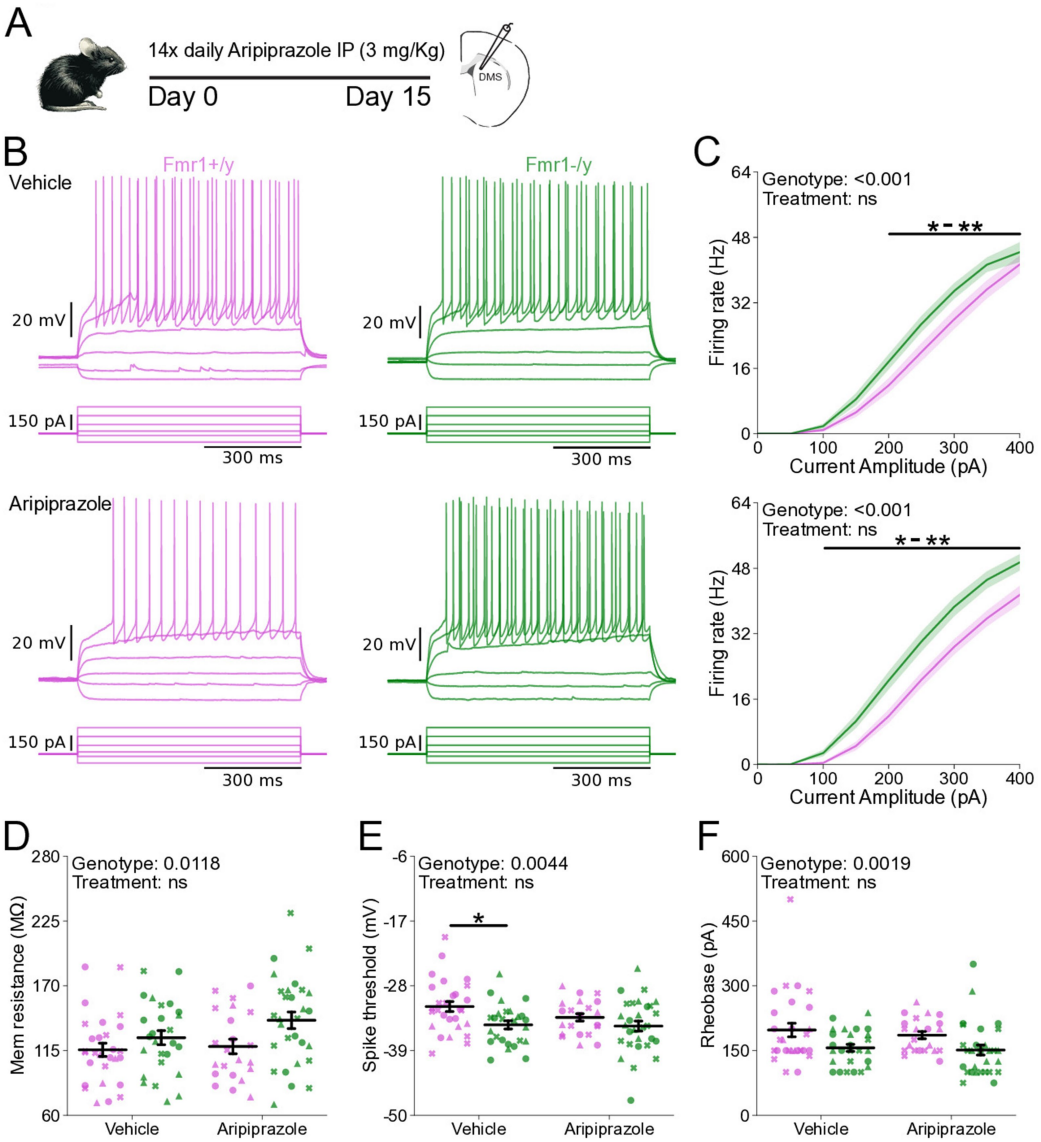
Chronic aripiprazole treatment does not ameliorate hyperexcitability in SPNs of *Fmr1−/y* mice. **(A)** Schematic showing daily intraperitoneal (IP) injections of aripiprazole (3 mg/kg) in adult mice for 14 days, followed by whole-cell recordings of SPNs in the DMS. **(B)** Representative current-clamp recordings showing action potential firing in response to current injections in D1-SPNs (top) and D2-SPNs (bottom) of *Fmr1+/y* (purple) and *Fmr1−/y* (green) P60 mice. n = 22 cells from N = 3 *Fmr1+/y* x Aripiprazole mice; n = 28 cells from N = 3 *Fmr1+/y* x Vehicle mice; n = 29 cells from N = 3 *Fmr1−/y* x Aripiprazole mice; n = 26 cells from N = 3 *Fmr1−/y* x Vehicle mice **(C)** Input–output relationship of firing frequency versus current amplitude (I-F curves) in SPNs from *Fmr1−/y* and *Fmr1+/y* mice treated with vehicle (top) or aripiprazole (bottom). **(D)** Average membrane resistance. **(E)** Average AP threshold **(F)** Average rheobase current. Plots show individual data points, with shape representing a mouse within the group (i.e., *Fmr1−/y* x D1-SPN) and the summary with mean ± SEM. *p < 0.05, **p < 0.01, ***p < 0.005, ****p < 0.001.

We next asked whether pharmacological interventions commonly used to manage symptoms in FXS influence SPN excitability. Aripiprazole is a second-generation antipsychotic that is prescribed to approximately ~30% of individuals with FXS to alleviate irritability, aggression and self-injurious behaviors (Eckert et al., 2019). Mice were treated with 3 mg/kg aripiprazole, a dose shown to reduce behavioral abnormalities induced by prenatal valproic acid exposure (Hara et al., 2017) and comparable to the human-equivalent dosage typically prescribed in FXS (Erickson et al., 2011; Eckert et al., 2019). To determine whether chronic aripiprazole treatment could rescue the hyperexcitability phenotype of adult DMS SPNs in *Fmr1−/y* mice, we performed whole-cell current-clamp recordings in acute brain slices of *Fmr1−/y* and *Fmr1+/y* mice following 14 days of daily treatment with either aripiprazole or saline (Figure 7A). As previously observed, SPNs of *Fmr1−/y* are hyperexcitable when compared to *Fmr1+/y* SPNs (Figure 7B). Aripiprazole treatment had no effect in this genotype difference, and the I-F relationship was unaltered and remained elevated in *Fmr1−/y* SPNs compared to *Fmr1+/y*. (Figure 7C; Genotype: *p* < 0.001, η^2^ = 0.125; Pulse Amplitude: *p* < 0.001, η^2^ = 0.907_;_ Genotype*Pulse Amplitude: *p* < 0.001, η^2^ = 0.0945). Multiple comparisons showed that the firing rate was increased in *Fmr1−/y* compared to *Fmr1+/y* from 200 pA to 350 pA in the Vehicle treated group and 100 pA to 400 pA in the Aripiprazole group (See Supplementary Table 1). We found no treatment differences in the I-F slope (Supplementary Figure S3L), the estimated maximum firing rate (Supplementary Figure S3M) or the current offset (Supplementary Figure S3N), except an increase in the estimated maximum firing rate of *Fmr1−/y* compared to *Fmr1+/y* in the vehicle group (Supplementary Figure S3M; Genotype: *p* = 0.00762, η^2^ = 0.0672, 95% CI [1.44, 9.19]; Post hoc comparison Vehicle: *p* = 0.044, 95% CI [−8.34, 2.32]; Aripiprazole: p = 0.260, 95% CI [1.81, 13.42]). Quantification of membrane resistance revealed a main effect of genotype with increased resistance in *Fmr1−/y* SPNs (Figure 7D; Genotype: *p* = 0.0118, η^2^ = 0.0596, 95% CI [−28.8, −3.69]; Post hoc comparison Vehicle: *p* = 0.43, 95% CI [−8.34, 2.32]; Aripiprazole: *p* = 0.08, 95% CI [1.81, 13.42]), consistent with our findings described in Figure 5. AP threshold was hyperpolarized in *Fmr1−/y* mice, but this difference was larger in the vehicle compared to the aripiprazole group (Figure 7E; Genotype: *p* = 0.00446, η^2^ = 0.0754, 95% CI [0.729, 3.86]; Post hoc comparison Vehicle: *p* = 0.026, 95% CI [0.91, 5.30]; Aripiprazole: *p* = 0.34, 95% CI [−0.65, 3.60]). However, the reduced effect in the aripiprazole group was due to a more hyperpolarized AP threshold in *Fmr1+/y* SPNs rather than a rescue of the deficit in the *Fmr1−/y* group. A similar pattern was observed for peak AHP voltage, which was strongly reduced in *Fmr1−/y* SPNs compared to *Fmr1+/y* under vehicle conditions, but less so after aripiprazole, due to a reduction in peak voltage in the WT group (Supplementary Figure S3B; Genotype: *p* = 0.0202, η^2^ = 0.0513, 95% CI [0.308, 3.56]; Post hoc comparison Vehicle: *p* = 0.023, 95% CI [0.91, 5.11]; Aripiprazole: *p* = 0.96, 95% CI [−1.54, 3.24]). Other AP properties such as AP half-width (Supplementary Figure S3C), maximum AP velocity (Supplementary Figures S3D,E), time of the maximum AP velocity (Supplementary Figure S3F), minimum AP velocity (Supplementary Figure S3G), time of minimum AP velocity (Supplementary Figure S3H), peak AP voltage (Supplementary Figure S3I), as well as resting membrane potential (Supplementary Figure S3J) and the time at which the peak AHP occurred (Supplementary Figure S3K) showed no statistically significant difference between genotypes when analyzed in combined SPN populations. In addition, rheobase was unaffected by aripiprazole (Figure 7F), with reduced values in *Fmr1−/y* SPNs persisting after treatment. Together, these findings indicate that chronic aripiprazole treatment does not significantly alter the intrinsic properties or AP kinetics of DMS SPNs in either *Fmr1+/y* or *Fmr1−/y* mice.

## Discussion

Here, we characterized the developmental trajectory of SPN dysfunction in the DMS of *Fmr1−/y* mice, a widely used genetic model of FXS. Our findings reveal hyperexcitability of both D1- and D2-SPNs in adult *Fmr1−/y* male mice, with stronger effects in D1-SPNs (Figures 5,6). In contrast, no such deficits were observed at P14–P15 (Figures 2,3), indicating that SPN excitability changes arise only after early postnatal development. We also found no significant differences in mEPSC frequency or amplitude in both SPN subtypes at P14-P15 (Figure 1) or P60 (Figure 4), suggesting normal glutamatergic synaptic inputs and consistent with previous observations of unaltered dendritic spine density in DMS SPNs (Huebschman et al., 2022). However, a different study has reported increased dendritic spine density and elevated mEPSC frequency in D1-SPNs of the DLS in *Fmr1−/y* mice (Longo et al., 2023), suggesting region-specific abnormalities of SPN synaptic properties across the striatum.

Hyperexcitability of DMS D1-SPNs was marked by a left-shifted I–F relationship, lower rheobase current, and broader APs with slower repolarization kinetics (Figure 5). Membrane resistance was elevated at the group level, but pairwise comparisons did not withstand correction and should be interpreted with caution (Figure 5C). In D2-SPNs, hyperexcitability was more modest and limited to a significant leftward shift in the I–F relationship. Similar abnormalities in excitability and AP kinetics have been observed in other neuron types of adult *Fmr1−/y* mice, including pyramidal neurons of the mPFC and hippocampus. Interestingly, these changes are cell type-specific and caused by distinct ion channel alterations. In the hippocampus, CA3 pyramidal neurons exhibit wider APs due to reduced BK channel function (Deng et al., 2013), whereas CA1 neurons show hyperexcitability and abnormal expression of dendritic HCN channels (Brager et al., 2012; Booker et al., 2020). In the cortex, L2/3 pyramidal neurons exhibit shorter APs with faster decay, driven by enhanced A-type (Kv4) potassium currents (Routh et al., 2017), whereas extratelencephalic, but not intratelencephalic L5 pyramidal neurons exhibit a hyperpolarized AP threshold, caused by reduced HCN and Kv1 currents (Kalmbach et al., 2015). These findings highlight complex, cell type-specific patterns of K^+^ channel dysfunction in *Fmr1−/y* mice, leading to distinct alterations in excitability and AP generation and kinetics across different brain regions. The differential severity between SPN subtypes is consistent with previous studies reporting distinct phenotypes in D1- and D2-SPNs (Longo et al., 2023; Giua et al., 2023). However, in contrast to our findings in the DMS, SPNs in the NAc exhibit opposite excitability adaptations, highlighting region-specific differences in *Fmr1*-associated deficits (Giua et al., 2023). The broader AP waveforms observed in DMS SPNs (Figure 6) suggest slower repolarization. Since BK channels facilitate rapid AP repolarization and Kv4 channels regulate subthreshold excitability, their dysfunction could cause slow AP decay without affecting threshold properties. Increases in AP width might also increase calcium influx in presynaptic terminals (Deng et al., 2013), suggesting that SPN synaptic output might be increased due to increased AP width and duration. Heightened excitability and output of D1-SPNs, coupled with more modest changes in D2-SPNs, could bias striatal activity toward excessive activation of the direct pathway, thereby disrupting normal activity patterns in frontostriatal circuits (Lee et al., 2016; Aoki et al., 2019; Janeček et al., 2025). Such a shift in striatal output may contribute to hallmark symptoms of FXS, including motor coordination deficits, impaired cognitive flexibility, hyperactivity, and impulsivity (Menon et al., 2004; Bagni et al., 2012; Wolff et al., 2013; Eckert et al., 2019; Usher et al., 2020). Future work identifying the precise channel alterations in SPNs, and determining how they affect SPN excitability, AP kinetics and overall striatal output will be important for understanding the mechanisms underlying DMS circuit dysfunction in *Fmr1−/y* mice.

Perhaps the most critical finding of this study is that SPN hyperexcitability in *Fmr1−/y* mice emerges only after early postnatal development. At P14-P15, SPNs exhibit normal intrinsic properties, with hyperexcitability detected only in adulthood (Figure 5). This delayed onset suggests that increased excitability is unlikely to result solely from the absence of FMRP, since in rodents FMRP expression peaks in the striatum during perinatal periods (Gholizadeh et al., 2015). This phenotype has important implications for understanding motor and cognitive symptoms in FXS. The DMS is a central hub for goal-directed behavior and cognitive flexibility, and imbalances in SPN activity have been implicated in multiple neurodevelopmental disorders, including ASD (Shepherd, 2013; Fuccillo, 2016). A review of the developmental trajectory of behaviors in FXS reported that many symptoms change in severity throughout life (Usher et al., 2020; Cregenzán-Royo et al., 2022). The delayed onset of SPN hyperexcitability may therefore parallel the developmental trajectory of these behavioral symptoms, raising the possibility that striatal adaptations contribute to their age-dependent differences. Future studies should examine how changes in striatal activity and connectivity relate to the progression of maladaptive behaviors across development. Our findings also have therapeutic relevance. Aripiprazole, which is widely prescribed to manage irritability and aggression in FXS (Dominick et al., 2021), did not alter the hyperexcitability of DMS SPNs in *Fmr1−/y* mice following chronic treatment (Figure 7). This suggests that its clinical benefits may arise through mechanisms unrelated to SPN excitability. Given the substantial adverse effects associated with aripiprazole (Marcus et al., 2009; Erickson et al., 2011), it will be important to further assess its impact on striatal circuits and to pursue alternative therapeutic approaches that directly target core SPN dysfunction. One promising avenue involves positive allosteric modulators (PAMs) of the muscarinic acetylcholine M4 receptor, which restore synaptic plasticity deficits in SPNs of *Fmr1−/y* mice (Longo et al., 2023). Whether M4R PAMs can also normalize SPN excitability differences remains to be determined.

It is also important to consider the possibility that changes in SPN excitability represent a secondary adaptation to circuit-level disruptions induced by loss of FMRP. *Fmr1−/y* mice exhibit reduced cortical activity during early postnatal stages (Gonçalves et al., 2013; Goel et al., 2018; Kourdougli et al., 2023), indicating that cortical dysfunction precedes the onset of SPN excitability deficits. Given the strong functional coupling between cortical and dorsal striatal circuits (Fuccillo, 2016; Peixoto et al., 2016, 2019; Janeček et al., 2025), reduced cortical activity could progressively drive compensatory adaptations in SPNs, ultimately resulting in hyperexcitability. Within this framework, the more pronounced increase in D1-SPN excitability relative to D2-SPNs could potentially contribute to restore normal activity levels in frontostriatal networks, since D1-SPN activation positively modulates cortical activity (Oldenburg and Sabatini, 2015; Lee et al., 2016; Aoki et al., 2019; Janeček et al., 2025). A comparable mechanism has been observed in hippocampal CA1 neurons of *Fmr1−/y* mice, where reduced synaptic input is offset by increased membrane excitability, thereby maintaining normal input–output function (Booker et al., 2020). Consistent with this model, both D1- and D2-SPNs show normal recruitment during self-initiated locomotion in adult *Fmr1−/y* mice, suggesting that striatal activity is normalized, at least in some behavioral contexts (Longo et al., 2023). Input-dependent adaptations may also explain why D1-SPNs exhibit a larger increase in excitability compared to D2-SPNs, since D2-SPNs are intrinsically more excitable than D1-SPNs and might be less sensitive to reductions in glutamatergic drive. Alternatively, these excitability changes in SPNs might be induced by local dysfunction of striatal circuits caused by impaired local interneuron activity or abnormal dopaminergic signaling, both of which are altered in *Fmr1−/y* mice (Goel et al., 2018; Kourdougli et al., 2023). Determining whether SPN hyperexcitability arises from cell-autonomous deficits caused by the loss of FMRP, or instead represents an adaptation to extrinsic circuit disruptions, will be critical for further understanding striatal dysfunction in FXS and guiding the development of effective therapeutic strategies.

While our study provides valuable insights into the developmental trajectory of SPN dysfunction in FXS, it also raises important questions that warrant further investigation. First, the molecular mechanisms driving SPN hyperexcitability remain unclear. It is also not known whether the excitability differences between SPN subtypes reflect variations in the magnitude of a shared pathophysiological process or arise from distinct mechanisms altogether. Second, our analysis of synaptic function was restricted to AMPAR-mediated mEPSCs. However, because mEPSC recordings primarily reflect postsynaptic AMPAR-mediated events from spontaneously released vesicles, they may not fully capture changes in evoked release probability or short-term plasticity. This approach also precludes direct comparison with previous studies that focused on dendritic spine morphology, and may mask input-specific abnormalities, such as potential disruptions in corticostriatal transmission. Addressing these gaps will be essential for achieving a more comprehensive understanding of DMS connectivity deficits in FXS. An additional caveat of our study relates to the classification of SPNs based on D1-Tom reporter expression. Although the vast majority of SPNs segregate into either the D1- or D2-expressing populations, a small subset of neurons co-express both receptors. The reported prevalence of such cells in dorsal striatum is relatively low (~5%) (Biezonski et al., 2015), but their existence introduces a potential margin of error in distinguishing between D1- and D2-SPNs in our dataset. However, our data show well-established excitability differences between D1- and D2-SPNs, supporting the validity of the D1-Tom labeling approach for distinguishing these populations. Finally, the broader functional consequences of SPN hyperexcitability for striatal circuit dynamics and behavior remain unclear. Given the early cortical disruptions reported in *Fmr1−/y* mice, future studies should aim to determine how these cortical abnormalities impact downstream striatal circuits *in vivo* and how such changes contribute to deficits in goal-directed behavior, motor control, and cognitive flexibility.

## Data Availability

The raw data supporting the conclusions of this article will be made available by the authors, without undue reservation.
