# Supplementary figures and images for: Delayed onset of striatal projection neuron hyperexcitability in Fmr1−/y mice
Source: Front Cell Neurosci. 2025 Nov 13;19:1667476. doi: 10.3389/fncel.2025.1667476 (PMC12657441; doi:10.3389/fncel.2025.1667476)

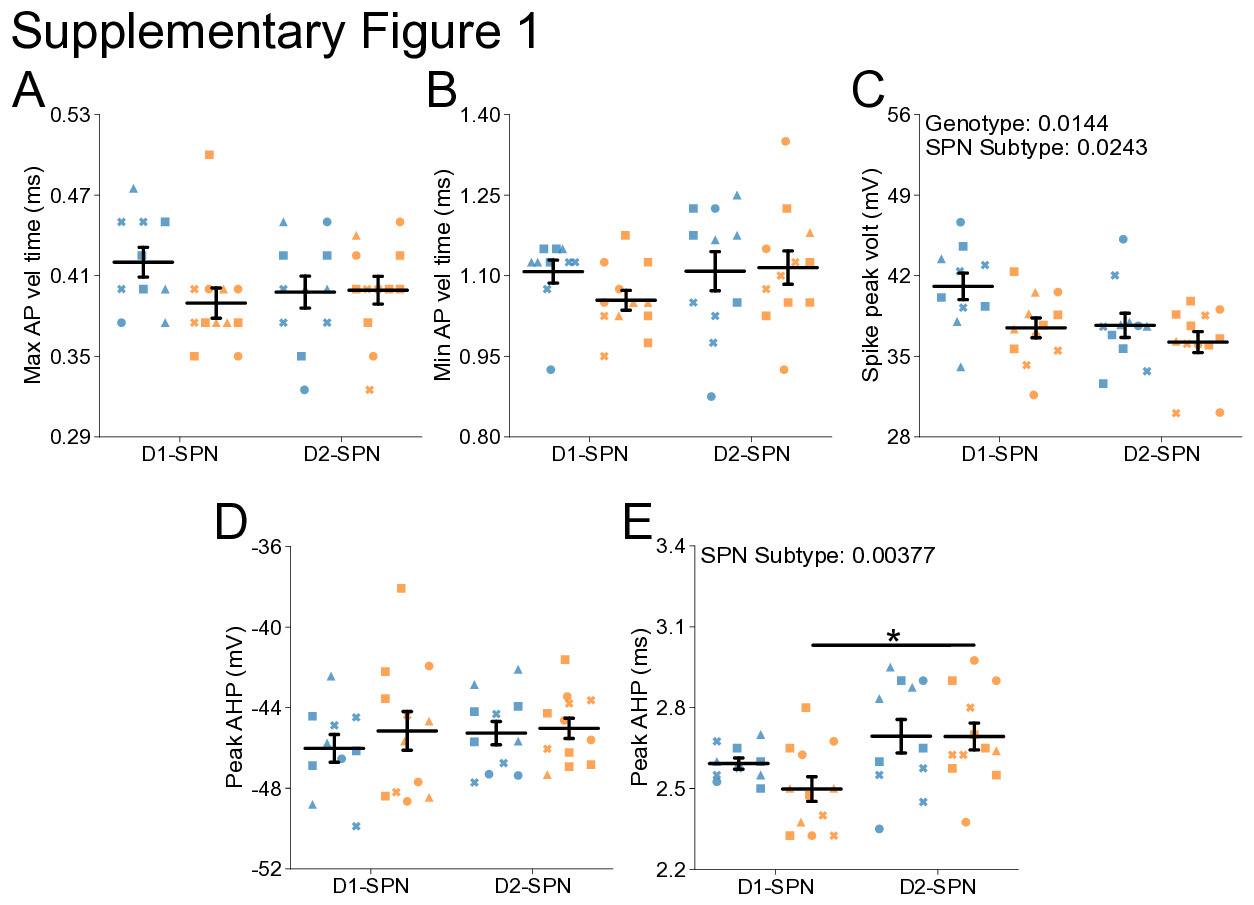

Supplement: Supplementary file 1 [file Figure_1.JPEG]

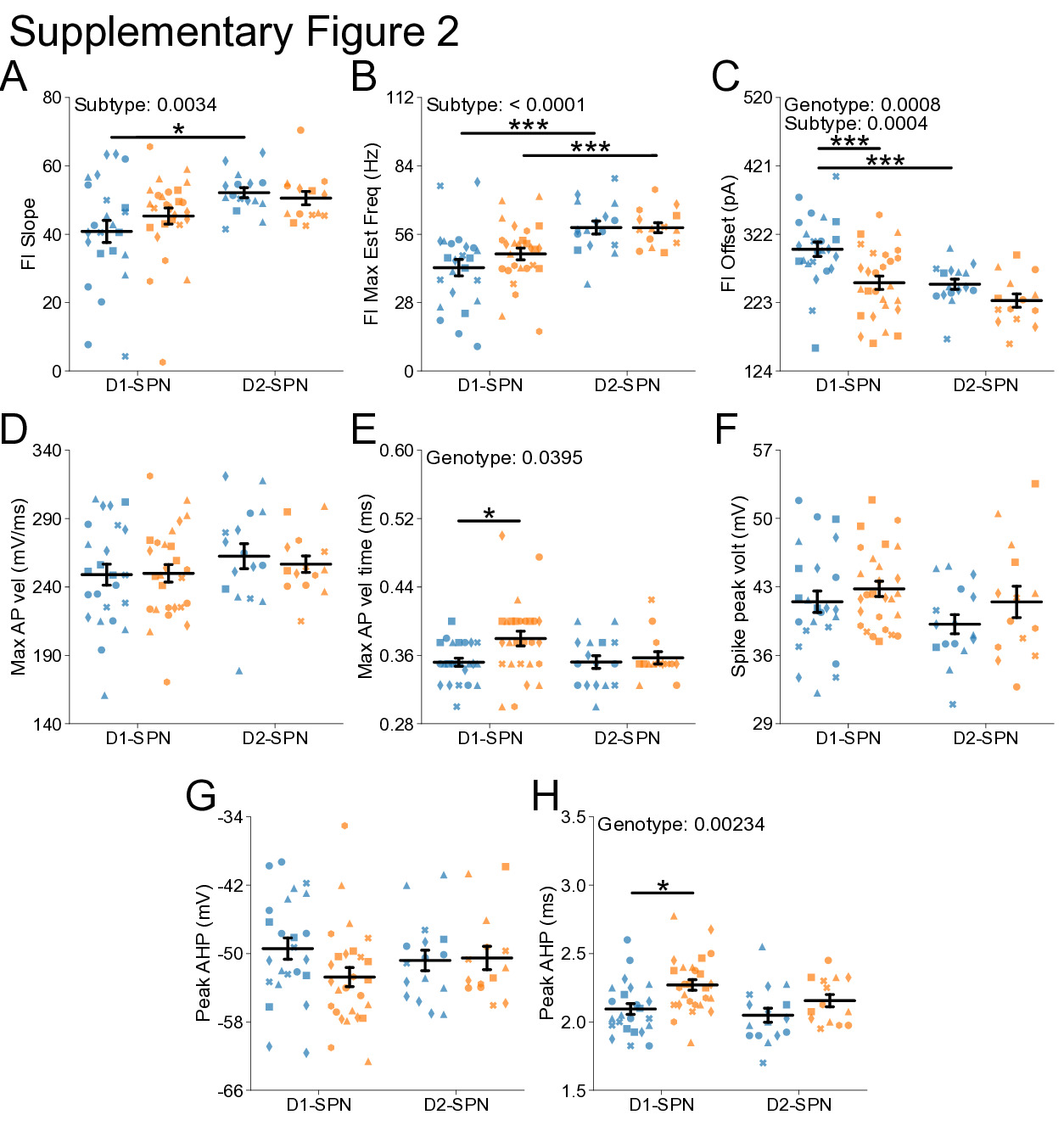

Supplement: Supplementary file 2 [file Figure_2.JPEG]

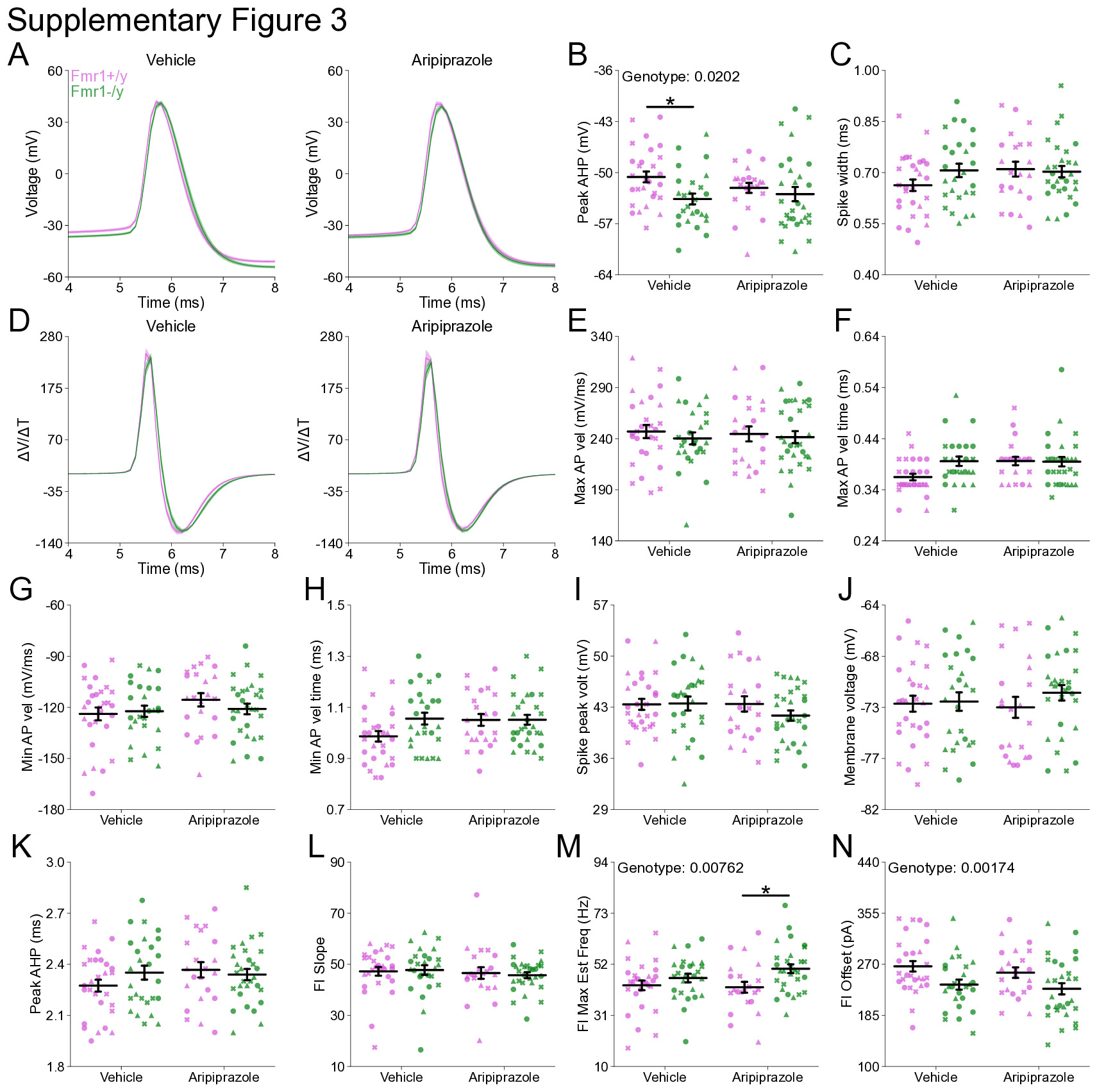

Supplement: Supplementary file 3 [file Figure_3.JPEG]
